# Excap: Maximization of Haplotypic Diversity of Linked Markers

**DOI:** 10.1371/journal.pone.0079012

**Published:** 2013-11-07

**Authors:** André Kahles, Fahad Sarqume, Peter Savolainen, Lars Arvestad

**Affiliations:** 1 KTH Royal Institute of Technology, Stockholm Bioinformatics Center, School of Computer Science and Communication, Stockholm, Sweden; 2 KTH Royal Institute of Technology, School of Biotechnology, Stockholm, Sweden; 3 KTH Royal Institute of Technology, Science for Life Laboratory, Department of Gene Technology, Stockholm, Sweden; 4 Swedish eScience Research Center, Department of Numerical Analysis and Computing Science, Stockholm University, Stockholm, Sweden; 5 KTH Royal Institute of Technology, Science for Life Laboratory, School of Computer Science and Communication, Stockholm, Sweden; Vanderbilt University Medical Center, United States of America

## Abstract

Genetic markers, defined as variable regions of DNA, can be utilized for distinguishing individuals or populations. As long as markers are independent, it is easy to combine the information they provide. For nonrecombinant sequences like mtDNA, choosing the right set of markers for forensic applications can be difficult and requires careful consideration. In particular, one wants to maximize the utility of the markers. Until now, this has mainly been done by hand.

We propose an algorithm that finds the most informative subset of a set of markers. The algorithm uses a depth first search combined with a branch-and-bound approach. Since the worst case complexity is exponential, we also propose some data-reduction techniques and a heuristic.

We implemented the algorithm and applied it to two forensic caseworks using mitochondrial DNA, which resulted in marker sets with significantly improved haplotypic diversity compared to previous suggestions. Additionally, we evaluated the quality of the estimation with an artificial dataset of mtDNA. The heuristic is shown to provide extensive speedup at little cost in accuracy.

## Introduction

Genetic markers are ubiquitous in molecular biology and have many applications, such as forensic analysis, taxonomic barcoding, and detection of inherited diseases. While full-length sequences would be the preferred material for most studies, real-world circumstances sometimes force the usage of a limited set of markers as a proxy. In particular, SNPs and short tandem repeats are commonly used as markers in forensics. How to choose markers is an important question and many factors can affect such a decision. For example, sample availability, application, sequencing technology, cost, time, and practicality has to be taken under consideration [1]–[4]. Although high-throughput sequencing has revolutionized molecular biology and genetics, it is not yet an economically feasible route for forensic laboratories.

The costs of the analysis and the amount of work are usually directly dependent on the number of markers that should be examined. This number is affected by marker length, which in turn depends on available sequencing technology and the size of the biological sample.

One specific goal is to maximize the information gained by a set of markers. A common measurement for the information gained from a marker is its *haplotypic diversity* 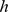 [5], [6]. It describes the probability that this marker differs in two individuals randomly chosen from a given population. Hence, 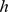 is a measurement of the genetic variability of the marker. In forensic sciences this diversity is known as *exclusion capacity*, because the markers are used to identify individuals or to exclude them from a panel of suspects.

If the markers are found in nuclear DNA, as is chosen for many applications, they can often be regarded as statistically independent, provided the markers are situated on different chromosomes or sufficiently far apart if on the same chromosome. Thus, the haplotypic diversity of a set of markers can be calculated by multiplying the marker's diversities.

In some applications, nuclear DNA is less interesting or unsuitable. In forensics, for example, nuclear DNA is for some sample types, such as hairs, often highly degraded and many markers may therefore not be available (see e.g. [7], [8]). Another example can be phylogenetic studies, where other DNA sources have properties more suitable for the investigation's purpose [9]. In both cases, DNA from mitochondria (mtDNA), which is many times more abundant, can be studied. However, a disadvantage with mtDNA is its relatively small size, and it has to be considered as one single linkage group. Thus, the information given by its potential markers may no longer be considered as statistically independent. This raises a question: if you are given an unbiased sample of mtDNA sequences from a population and want to find the most efficient combination of markers, what do you do?

Genetic variability is commonly due to a relatively small and dispersed set of positions, causing potential markers to be dispersed as well. If the variable positions, and hence the potential markers, are linked, the haplotypic diversity of marker sets cannot directly be determined from 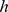-values of single markers. Thus, it is not easy to find the optimal subset of markers. To calculate the most informative marker subset, haplotypic diversities of the markers have to be estimated from a sample set of individuals taken from the population. Since the strength of the informational coupling between the single markers influences the haplotypic diversity of their combination, this has to be estimated from the given sample set as well. Today, this is done by a combination of visual inspection and subjective choice of markers in the lack of suitable programs. With this methodology it is very laborious and virtually impossible to find the best marker sets even for relatively small datasets.

We present algorithms for finding the most informative subset of markers, subject to constraints such as marker size and number, and estimating the markers' haplotypic diversity from a sample set given as multiple sequence alignment (MSA). We implemented the algorithms in the Excap program, which reads an input alignment in FASTA format and returns an optimal set of markers of a requested length and size. This is a significant step forward as compared to current practice.

## Materials and Methods

### Haplotypic diversity

The calculation of 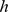 is based on an estimation for the diversity of a genetic marker [5]. Considering a sample set of size 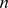 and a marker with 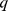 different haplotypes, each with frequency 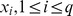, the haplotypic diversity is estimated as:
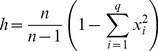
(1)


For technical reasons, a new method to calculate the diversity of a marker is introduced. Instead of the frequencies 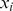, the number of sequences each haplotype comprises, 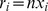, is used to count the number of possible pairs of different haplotypes in the sample, henceforth designated as *separation index* 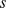:
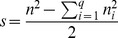
(2)


Its maximum 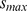 is 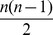 when the number of haplotypes equals the number of sequences (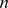) and therefore 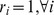. The haplotypic diversity can easily be calculated as
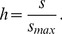
(3)


One advantage of this approach is that all 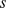-values can be handled as integers during the whole calculation without loss of precision. It furthermore simplifies the estimation of the maximum 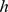-value a set of markers can achieve, which is used in our branch-and-bound algorithm.

### Data representation

**Figure 1:**
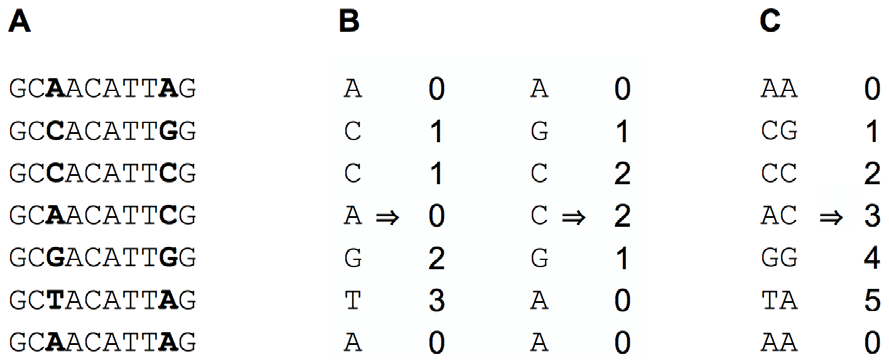
Data representation. Each polymorphic column is transformed into an integer vector. **a)** Multiple sequence alignment, polymorphic columns/markers in bold face. **b)** Haplocode representation of the markers. **c)** Haplocode representation of a combination of the markers.

The input data, given as an MSA, is reduced to its polymorphic columns. Each of those columns is transformed into an integer vector of length 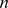, representing the different haplotypes. This vector is further designated as 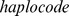. The different haplotypes are numbered from 0 to the number of haplotypes at this position minus 1. Figure 1 provides a small example.

A marker's *width* is the number of columns it spans in the MSA. Markers that are wider than 1 bp are allowed to overlap, but we ignore markers that are contained by another marker.

Analoguously to a marker, a set of markers has a haplocode describing its haplotypic information, too. The set's haplocode is easy to calculate in 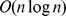 time by combining the haplocodes of the markers it contains. Furthermore, each marker 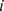 has a separation index 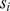 which indicates how many sequence pairs this marker separates. It is easy to calculate 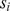 from the marker's haplocode in 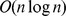 time. It is analogous to definite a separation index 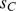 for a marker set 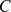.

Before combining single markers, the complexity of the dataset is reduced:

1. **Elimination of redundancy.** If two markers represent the same information or if the information of one marker is a subset of another marker's information, one of them is removed. The first case is easy to handle because markers that contain the same information will have the same haplocode. The second case is detected by looking at the haplocode of the two markers combined. If the combination's haplocode is identical to the haplocode of one of the markers, the other marker is deleted.

2. **Sorting of markers.** All markers are sorted by their separation index in decreasing order. This step is necessary for the estimation-step in the Excap algorithm.

### Finding the best marker set

Since the diversity of a marker set cannot be calculated from its members' diversities directly, all possible combinations have to be built up to determine their 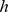-value. The number of possible combinations grows exponentially, and we will try to limit how many combinations we have to consider. Later on a heuristic is provided that gives a good approximation to the optimal solution.

To find the best marker set of size 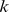, the sorted markers are combined successively using a depth-first search combined with a branch-and-bound approach. Beginning with the strongest marker, according to its separation index, the next markers with weaker 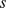
- values are added recursively step by step until the set has reached size 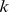. In each step it is estimated if the current set can achieve a better separation index than the best one found so far. If the separation index is worse, the recursion returns to the calling step and tries to add the marker with the next best 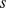-value to the set.

**Figure 2:**
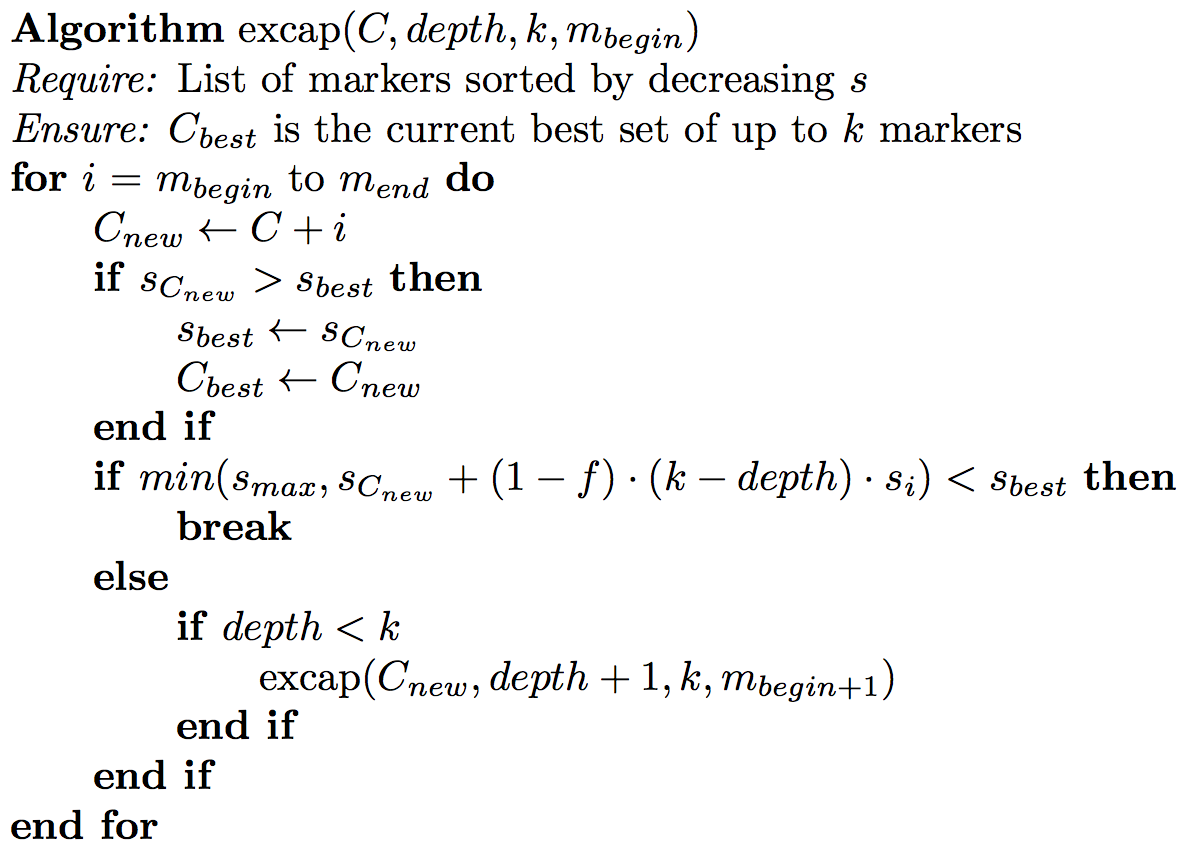
The excap algorithm.

The *excap* algorithm (Figure 2) describes how markers are combined using a depth-first search. The initial parameters are an empty set of markers (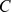), a pointer to the start of the list of sorted markers (*begin*), the maximum size of the set (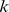) and the recursion depth (*depth*  =  1).

The worst case running time for finding the best combination of size 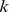 out of 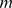 available markers is 
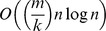
. To perform a little better for large values of 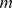 and 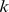, we provide a heuristic that modifies the estimation step and thereby carries out the bounding step earlier, see section.

### A bound for the separation index

In the bounding step, it is estimated how good a set of markers would be if 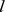 further markers were added before the set reached maximum size. If the set 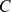 separates 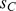 sequence pairs and if the current marker to add 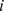 separates 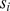 sequence pairs, then the combination of 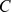 and 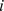 can at best separate 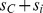 sequence pairs. That is a weak upper bound, relying on marker independence, but since the strength of the coupling of the single markers is unknown, it is difficult to estimate how much information they share. Since the markers are sorted decreasingly by their 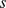-values, the separation index is bounded a set 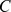 can achieve with 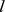 additional markers is bounded by
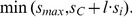
(4)


As a heuristic approach, we modified the bounding in order to reduce the number of alternatives. Instead of assuming that each newly added marker can contribute its whole separation index to the separation index of the new combination, the marker's 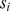 is scaled down by a factor 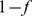, where 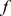 is the *heuristic parameter*. We require that 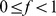, and 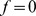 corresponds to the normal, non-heuristic approach, while 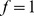 would mean that every added marker is assumed to contribute no new information. The separation index is heuristically bounded by
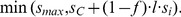
(5)


Note that 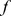 most likely has a non-linear effect on the method's estimation of 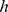, which is probably undesirable from a user's perspective.

### Artificial data

An artificial population was generated, represented by 10,000 sequences with a length of 16,000 bp each. Ten markers, each spanning 800 bp, with different sets of polymorphic columns and increasing 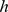-values were introduced in sequences. The mutations were made using the pseudo-random number-generator mt19937 [10] provided by the Boost C++ Library with default parameters.

From the given population, sample sets of different sizes have been drawn (uniformly at random, using mt19937).

### Biological data

The biological dataset consisted of 241 full length mtDNA sequences also used by Coble *et al* [11], which were taken from the mtDB database [12].

All sequences were aligned to the revised Cambridge reference sequence (rCRS) for human mtDNA [13] using the Kalign program [14] with its default values. In all calculations the rCRS was not considered as a part of the dataset and was only used for indexing.

This alignment induced 483 polymorphisms, and 264 remained after reducing redundancy (see Section ).

The 241 input sequences were separated in 18 groups according to their HV1/HV2 type based on the rules of Coble *et al*. Some calculations were limited to 59 significant positions from the coding region, previously defined by Coble et al.

The disease position exclusions were done according to the information available in the Mitomap project [15].

## Results

### Artificial data

To test the accuracy of the estimation and the influence of the sample size on the quality of the estimated 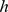-values for a given population an artificial population was created.

All 10,000 sequences were used as input for the Excap program in order to calculate the ``true'' 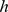-values of markers in the whole population. In the following step the haplotypic diversity of those markers should be estimated from samples randomly drawn from the population. This estimation was done using the Excap program. The procedure was repeated 100 times for each sample size (except for sample size 1,000 which was drawn 50 times). From each drawn set the markers' diversity in the whole population was estimated. The mean value and standard deviation of 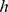 over all 100 (50) samples was recorded for each specific sample size.

**Figure 3:**
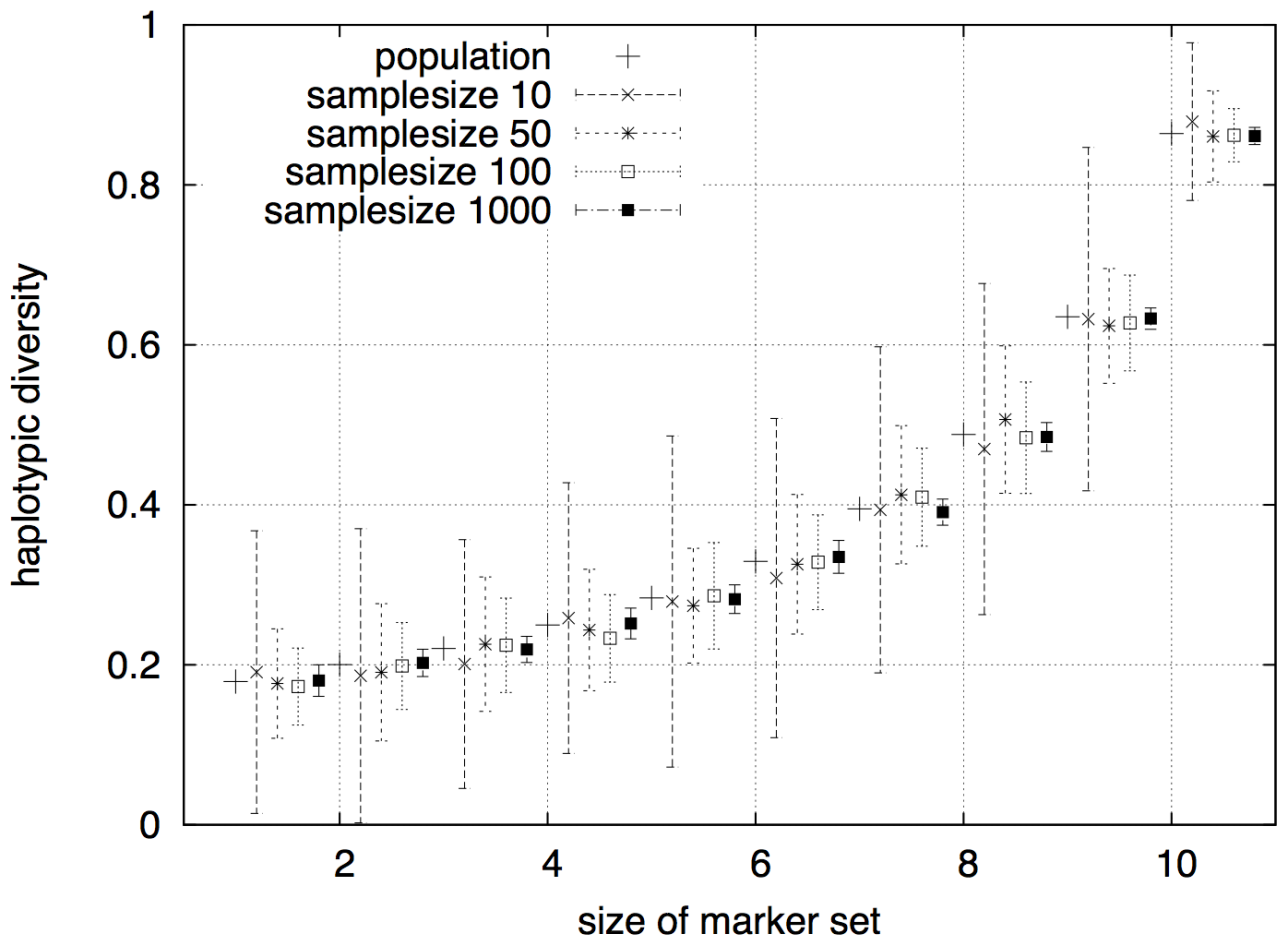
Influence of sample size. The estimated heterogozities and the corresponding standard deviations for sample sizes 10, 50, 100 and 1000.

The tests revealed a strong correlation between sample size and quality of the estimation, see Figure 3. For weak markers that had an 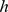-value below 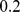, a sample set of size 10 was of limited value due to the high the standard deviation. With a sample size of 50 and stronger markers, the influence of the standard deviation sank and the estimates became more reliable. For a sample of size 10 and a marker with a low 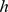-value (e.g. 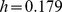) the standard deviation (0.177)was almost as high as the estimated value (0.191) itself and the marker's utility was therefor relatively uncertain. On the other hand, a sample size of 50 and a stronger marker (e.g. 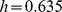) had an acceptable standard deviation (0.072).

It is clear that one must be careful not to overstate the importance of 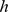 values estimated from small samples. It is worth noting, however, that standard deviation seems to be independent of 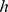 and only determined by sample size.

Detailed information about the correlation between sample size and the quality of the estimation can be found in Table 1.

**Table 1:** Correlation between sample size and quality of the estimated 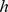-values.

		Estimates for different sample sizes			
Region	True 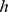	10	50	100	1000
1	0.18	0.19 (0.18)	0.18 (0.07)	0.18 (0.05)	0.18 (0.02)
2	0.20	0.19 (0.18)	0.19 (0.09)	0.20 (0.05)	0.20 (0.02)
3	0.22	0.20 (0.16)	0.23 (0.08)	0.22 (0.06)	0.22 (0.02)
4	0.25	0.26 (0.17)	0.24 (0.08)	0.23 (0.05)	0.25 (0.02)
5	0.28	0.28 (0.21)	0.27 (0.07)	0.29 (0.07)	0.28 (0.02)
6	0.33	0.31 (0.20)	0.33 (0.09)	0.33 (0.06)	0.33 (0.02)
7	0.39	0.39 (0.20)	0.41 (0.09)	0.41 (0.06)	0.39 (0.02)
8	0.49	0.47 (0.21)	0.51 (0.09)	0.48 (0.07)	0.48 (0.02)
9	0.64	0.63 (0.21)	0.62 (0.07)	0.63 (0.06)	0.63 (0.01)
10	0.86	0.88 (0.10)	0.86 (0.06)	0.86 (0.03)	0.86 (0.01)

The second column contains the 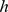-values of the population, which were estimated from samples (10, 50, 100 and 1000 individuals) using the Excap algorithm. The values in parentheses are the standard deviations of the estimated values.

### Tests on biological data

Tests on biological data was a comparison between different panels of mitochondrial single nucleotide polymorphisms (SNP panels) from the work of Coble *et al.* [11] and sets of SNPs created using the Excap program.

Coble *et al.* [11] sequenced the mtDNA of 241 individuals of and presented eight different multiplex panels of overall 59 SNPs which were designed to complement the results of the HV1/HV2 testing. The panels were chosen to maximize their ability to separate two individuals from the same subtype. Due to practical reasons, Coble *et al* [11] excluded all polymorphic positions related to diseases or positions with nonsynonymous mutations. All positions of the hypervariable regions were also disregarded.

To re-evaluate the eight panels, the sequences from their respective HV1/HV2 types, as designated by [11], were given as input to Excap. That is, if panel B was most applicable to HV1/HV2 types H:2, H:3 and H:6, Excap was run on the sequences of those subtypes. To keep our results comparable, only the 59 positions used in the multiplexed panels proposed by Coble have been taken as input data for the recalculation. Would Excap suggest the same marker sets, or find better combinations?

We found that Excap gave better marker sets for each of the eight panels, either by having a higher haplotypic diversity with the same number of markers (seven cases) or by having a smaller marker set (on case) but the same 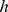. The improvements on diversity ranged from 0 % (but less SNPs used) to 101.81 %. See Table 2 for all results. The actual differences in terms of chosen SNPs are shown in Table 3 and they demonstrate why the automated Excap method does better than visual inspection based on individual sites' diversity. Also note that the actual number of different SNP positions are reduced from 59 to 47.

**Table 2:** Comparison of multiplexed SNP panels I.

Panel	Group	Size	Achieved 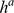	Size 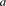	Diff	Improvement (%)
A	H:1	32	0.90 (0.89)	11 (11)	0.01	**1.21**
B	H:2, H:3, H:6	48	0.91 (0.90)	11 (11)	0.01	**0.62**
C	V:1, H:5	38	0.84 (0.81)	10 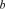 (11)	0.03	**2.95**
D	J:1, J:2, K:2, K:3	38	0.89 (0.78)	10 (10)	0.11	**14.45**
E	J:4, T:2, T:3, H:4	35	0.87 (0.82)	7 (7)	0.05	**6.32**
F	V:1, H:1, H:2, H:3	93	0.91 (0.45)	10 (10)	0.46	**101.81**
G	J:1, J:3, T:1	50	0.86 (0.74)	11 (11)	0.12	**17.32**
H	K:1	15	0.87 (0.87)	6 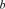 (7)	0.00	**0.00** 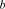

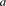
 The values in parenthesis are the results from Coble *et al*.

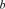
 This is the best possible result for the given input data — all individuals could be singled out.

The polymorphic positions combined by the algorithm were limited to the 59 SNPs which were part of Coble's presented multiplexed panel. The size of a haplogroup refers to the number of sequences in it.

**Table 3:** Improved panels for HV subtypes.

A	B	C	D	E	F	G	H
H:1	H:2, H:3, H:6	V:1, H :5	J:1, J:2, K:2, K:3	J:4, T:2, T:3, H:4	V:1, H:1, H:2, H:3	J:1, J:3, T:1	K :1
72 (0.12)	477 (0.12)	72 (0.50)	482 (0.24)	3010 (0.51)	72 (0.39)	482 (0.12)	64 (0.14)
477 (0.18)	3010 (0.29)	513 (0.11)	3010 (0.48)	4808 (0.11)	477 (0.10)	3010 (0.50)	4688 (0.14)
3010 (0.47)	3915 (0.12)	4580 (0.47)	5198 (0.15)	5147 (0.11)	3010 (0.32)	3826 (0.08)	11377 (0.44)
4793 (0.32)	4745 (0.08)	5250 (0.11)	6260 (0.15)	9380 (0.11)	3915 (0.06)	3834 (0.08)	13293 (0.26)
5004 (0.12)	5004 (0.29)	11377 (0.05)	6293 (0.05)	9899 (0.40)	4793 (0.12)	5198 (0.08)	14305 (0.44)
7202 (0.12)	6776 (0.39)	11719 (0.11)	9548 (0.15)	15067 (0.21)	6776 (0.20)	6293 (0.15)	16519 (0.14)
10211 (0.18)	8592 (0.16)	12438 (0.15)	11485 (0.35)	16519 (0.26)	10211 (0.08)	7891 (0.08)	
11377 (0.06)	10394 (0.23)	14770 (0.15)	12858 (0.05)		10394 (0.14)	11533 (0.08)	
12858 (0.12)	10754 (0.12)	15833 (0.32)	15355 (0.24)		12438 (0.06)	12795 (0.04)	
14470 (0.12)	14560 (0.08)	16519 (0.41)	16519 (0.50)		16519 (0.41)	15043 (0.08)	
16519 (0.23)	16519 (0.37)					16519 (0.42)	

*Positions used by Coble et al., but ignored here*							
4580 (0.12)	11864 (0.08)	12810 (0.11)	9635 (0.11)	11914 (0.06)	64 (0.02)	4688 (0.08)	12795 (0.27)
7028 (0.12)	15340 (0.08)	15884 (0.05)	11914 (0.05)		4745 (0.06)	12007 (0.04)	
			15884 (0.05)		10685 (0.04)	16390 (0.04)	
			16368 (0.11)		11377 (0.04)		
					14560 (0.04)		
					14869 (0.04)		
					14470 (0.06)		
					16390 (0.02)		

The modified panels A thorugh H from 2, as suggested by Excap. For each haplogroup, the suggested SNP are listed, with their corresponding 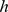 (when analyzed independently). Underlined SNPs are those that were introduced by Excap and the lower part of the table contains SNPs that were excluded by Excap but suggested by Coble *et al*. The data shows how some SNPs have a high 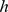, but are not informative in combination with other SNPs. For example, panel H where 12795 has 4th highest 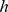, but it does not contribute at all towards a better combined 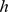.

Even better results were achieved with the input data not limited to the 59 SNPs present in Coble's panels. To achieve similar initial conditions, all positions of the hypervariable regions (73-340 and 16024-16365), the poly AC repeat (515-524) and all positions related to diseases (174 positions) were excluded using information provided by the MitoMap database [15]. Input data was the same sets of sequences as used for the previous calculations. All panels could be improved significantly (see Table 4).

**Table 4:** Comparison of multiplexed SNP panels II.

Panel	Achieved 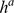	Size	Diff	Improvement (%)
A	0.93 (0.89)	11	0.04	**4.02**
B	0.92 (0.90)	11	0.02	**2.05**
C	0.90 (0.81)	11	0.09	**10.89**
D	0.92 (0.78)	10	0.15	**18.69**
E	0.87 (0.82)	7	0.05	**6.75**
F	0.91 (0.45)	10	0.46	**101.81**
G	0.87 (0.74)	11	0.13	**18.71**
H	0.90 (0.87)	7	0.03	**3.80**

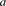
 The values in parenthesis are the results from Coble *et al*.

For this calculations HV1, HV2, the poly AC region and all positions related to diseases were excluded.

**Figure 4:**
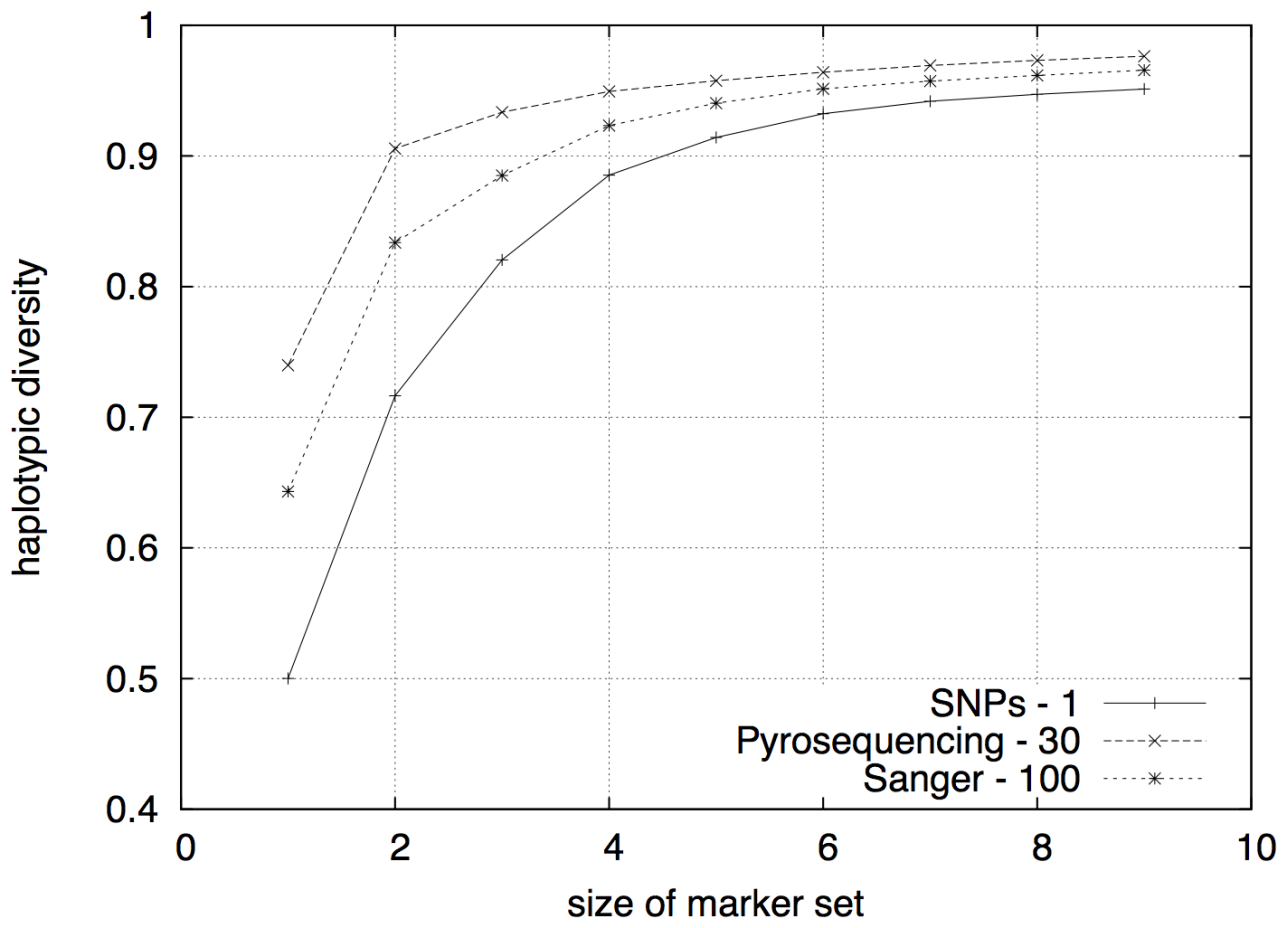
Estimated heterogozities. The more markers a combination contains the less effect the width has to the overall information of the combination.

Another important result is the correlation between the haplotypic diversity and the width of the combined markers in biological data. We computed 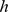-values for up to 9 markers f widths 1, 30 or 100 bp, on mitochondrial genome sequences. As few as three SNPs, or two 30 bp markers, have a higher haplotypic diversity than one 100 bp marker, see Figure 4. Also note that to get the same 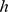 as from nine SNPs, you would need as much as four 100 bp markers or six 30 bp markers.

With increasing set size 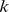, the 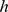-value of a set converges to a maximum, defined as the diversity of the whole sequence. The size of the marker set has a higher effect to the convergence than the width of the included markers and the speed of convergence decreases with the growing set size. This has an important effect on practical work because, e.g., typing 20 SNPs instead of 10 SNPs may be much more expensive, but does not really provide more information.

### Heuristic analysis

Running Excap on large datasets can be time consuming and a heuristic approach becomes necessary. To get a better understanding of our heuristic's impact on results and execution times, we again turned to the data from [11].

We ran the Excap algorithm to find an optimal marker set of 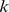 SNPs with different values on the heuristic parameter 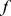, starting from 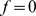 (no heuristic) and up to 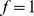. Each calculation was performed for a single SNP-marker up to a set of seven SNP-markers.

**Figure 5:**
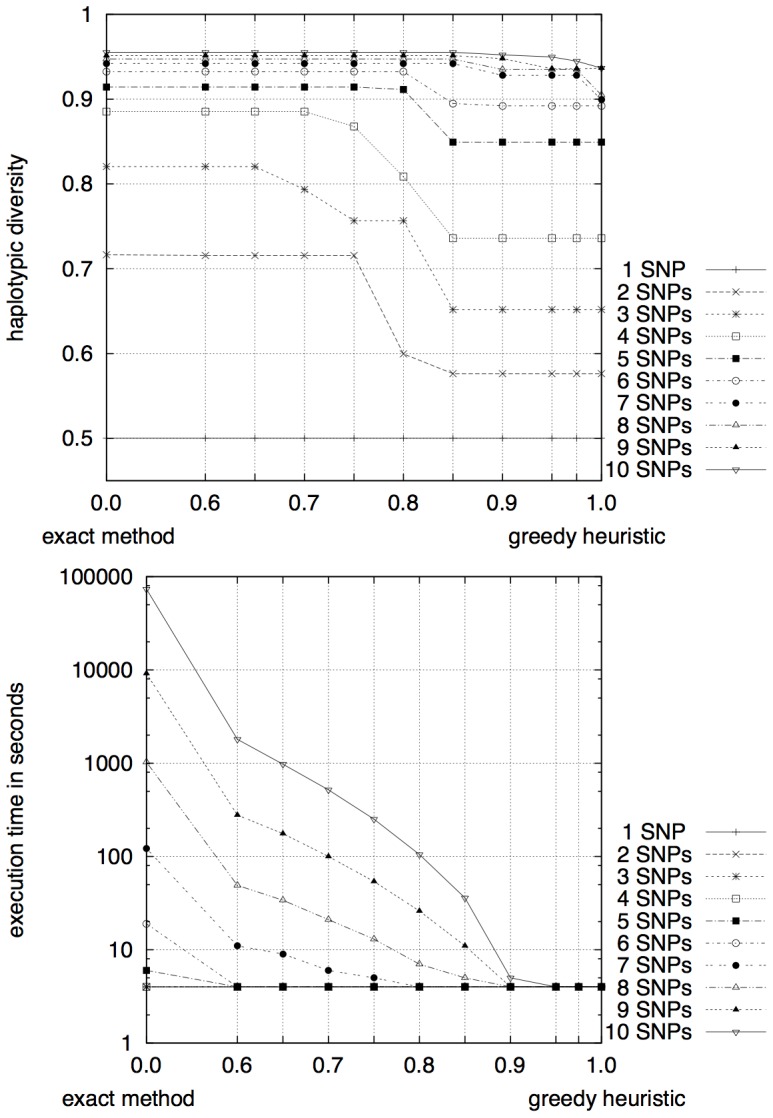
Marker set size and 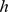. The more markers a combination contains and the more variable the combinations get, the more stable are the estimations also under a stronger heuristic.

The more markers a set contained, with consequently higher 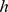, the more stable the estimation of 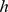 was, see Figure 5(a). For a set of four markers, 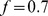 still resulted in the optimal solution. For 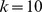, we could set 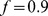 without notably worse estimation of 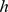. This observation is encouraging, since it is for the larger values on 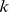 that the heuristic becomes indispensable.

In the tested cases, the heuristic approach had a substantial improvement of execution time. With increasing 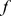, the execution time dropped exponentially, see Figure 5(b). Calculating the optimal set of seven SNPs in the data provided by Coble *et al.* took 143 seconds without heuristic. Using a 80 % heuristic reduced runningtime to 5 seconds and the provided solution was still the optimal one. The most dramatic case is for 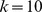, which dropped from being a day-to-day calculation to executing in a few seconds when 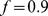. As a comparison with Figure 5(c) reveals, 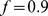 gives a result which is still close to optimal.

## Discussion

Our experiments with Excap on artificial and biological data showed significant advantages. Most sets of variable markers chosen by hand are not optimal. In almost every case, the most variable markers do not form the most variable *set*. In many cases, one marker with a weak diversity improved the chosen set significantly, much more than a marker with a much higher 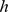-value which is not easy to find with a ``manual'' approach. Although our main algorithm's execution time is exponential, the related heuristic achieves reasonable and competitive results in many practical cases. Importantly, our parameterized heuristic allows for experimentation and adaption to different datasets.

As long as there is a dataset available that is big enough and representative for a certain population, Excap can be used to estimate the diversity and to optimize the choice of markers. One should be aware to keep the size and quality of the provided sample set in mind and not to overestimate the results.

There are some important consequences arising from having our program suggesting markers. Besides manual labour being reduced, the risk of making mistakes decreases significantly. Achieving the same variability with less and shorter markers also results in a reduction of costs. It is clear that the quality of chosen markers improves and that data is utilized better.

There is an important cost-benefit analysis to do when making decisions on marker sets, since there are limits of utility to including yet another marker. Excap enables a careful consideration regarding how many markers to use by estimating how much more information can be gained.

We believe Excap will also help choosing between technologies for DNA typing, and not just what markers to choose. By systematically trying different marker sizes and set sizes, one can determine the most economical and efficient way to reach a desired haplotypic diversity. As shown in Figure 4, a small number of SNPs may be more informative than hundreds of bases in larger markers.

There are opportunities for improvements to the present work. The suggested heuristic was a first approach to keep computation time in manageable scales, but is not necessarily the most efficient one. It could also be worthwhile to analyze the influence of the heuristic factor 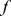 on the quality of the estimated 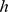-value. A heuristic factor 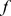 effecting the results in a more sensitive way than the linear one might allow a more fine-grained application of the heuristic. Due to the simple heuristic approach it is possible that a heuristic with a smaller 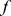-value results in a worse set of markers than one with a greater 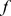. We would also like to investigate an approach in which the heuristic was used as a preprocessing step to reduce a large set of input markers. The optimal algorithm could then be applied to this more manageable set of markers. Furthermore, it could be investigated how existing parametrized algorithms, that solve NP-complete problems for a fixed parameter in polynomial time, could be applied to this problem. The size of the optimal set could be such a fixed parameter.

### Availability

Excap is written in C++ and the source code is hosted at http://sourceforge.net/projects/excap, distributed under the GNU Public License.
